# Temperature- and light stress adaptations in Zygnematophyceae: The challenges of a semi-terrestrial lifestyle

**DOI:** 10.3389/fpls.2022.945394

**Published:** 2022-07-19

**Authors:** Charlotte Permann, Burkhard Becker, Andreas Holzinger

**Affiliations:** ^1^Department of Botany, Functional Plant Biology, University of Innsbruck, Innsbruck, Austria; ^2^Department of Biology, Institute for Plant Sciences, University of Cologne, Cologne, Germany

**Keywords:** abiotic stress, Antarctic, arctic, climate change, cold stress, heat stress, ultraviolet radiation

## Abstract

Streptophyte green algae comprise the origin of land plants and therefore life on earth as we know it today. While terrestrialization opened new habitats, leaving the aquatic environment brought additional abiotic stresses. More-drastic temperature shifts and high light levels are major abiotic stresses in semi-terrestrial habitats, in addition to desiccation, which has been reviewed elsewhere. Zygnematophyceae, a species-rich class of streptophyte green algae, is considered a sister-group to embryophytes. They have developed a variety of avoidance and adaptation mechanisms to protect against temperature extremes and high radiation in the form of photosynthetically active and ultraviolet radiation (UV) radiation occurring on land. Recently, knowledge of transcriptomic and metabolomic changes as consequences of these stresses has become available. Land-plant stress-signaling pathways producing homologs of key enzymes have been described in Zygnematophyceae. An efficient adaptation strategy is their mat-like growth habit, which provides self-shading and protects lower layers from harmful radiation. Additionally, Zygnematophyceae possess phenolic compounds with UV-screening ability. Resting stages such as vegetative pre-akinetes tolerate freezing to a much higher extent than do young cells. Sexual reproduction occurs by conjugation without the formation of flagellated male gametes, which can be seen as an advantage in water-deficient habitats. The resulting zygospores possess a multilayer cell wall, contributing to their resistance to terrestrial conditions. Especially in the context of global change, understanding temperature and light tolerance is crucial.

## Streptophyte green algae and the origin of land plants

The exploitation of terrestrial environments as a new habitat and the consequent evolution of land plants (Embryophta) has been studied for decades. Approximately 1 billion years ago, the two sister clades Chlorophyta and Charophyta were formed by the split of Chloroplastida (Leliaert et al., 2012; Becker, 2013; de Vries and Archibald, 2018). While both clades contain taxa that are adapted to terrestrial environments (Holzinger and Karsten, 2013), embryophytes emerged within the group of Charophyta (streptophyte algae) as shown by expressed sequence tags and transcriptome data (Wodniok et al., 2011; Becker, 2013; Leebens-Mack et al., 2019). These streptophyte green algae are a complex and diverse paraphyletic group, ranging from unicellular flagellates to branching filaments (Leliaert et al., 2012). They comprise two paraphyletic main groups, the basal branching KCM-grade (Klebsormidiophyceae, Chlorokybophyceae, and Mesostigmatophyceae) and the later-branching ZCC-grade (Zygnematophyceae, Coleochaetophyceae, and Charophyceae; de Vries et al., 2016). While many species occur primarily in freshwater habitats, terrestrial forms have been reported in Chlorokybophyceae (Lewis and McCourt, 2004), Klebsormidiophyceae (Karsten et al., 2010; Karsten and Holzinger, 2012), and Zygnematophyceae (Lewis and McCourt, 2004; Holzinger et al., 2009, 2010). Of these, the class Zygnematophyceae has been established as a sister lineage to embryophytes, despite their simple body plan compared to other streptophyte green algae (de Vries and Archibald, 2018; Leebens-Mack et al., 2019). The first full zygnematophycean genomes of *Spirogloea muscicola* and *Mesotaenium endlicherianum* have been published just recently, as well as a draft genome of *Penium margaritaceum* (Cheng et al., 2019; Jiao et al., 2020). Recent studies have also shown that Zygnematophyceae possess hallmark genes (e.g., *GRAS*, *PYR/PYL/RCAR*, and plastid-associated genes) also involved in the stress response of land plants (de Vries et al., 2018; Cheng et al., 2019; Schreiber et al., 2022). Moreover, they are the only members of the charophycean green algae (CGA group), with land-plant cellulose synthase orthologs (Fitzek et al., 2019).

**FIGURE 1:**
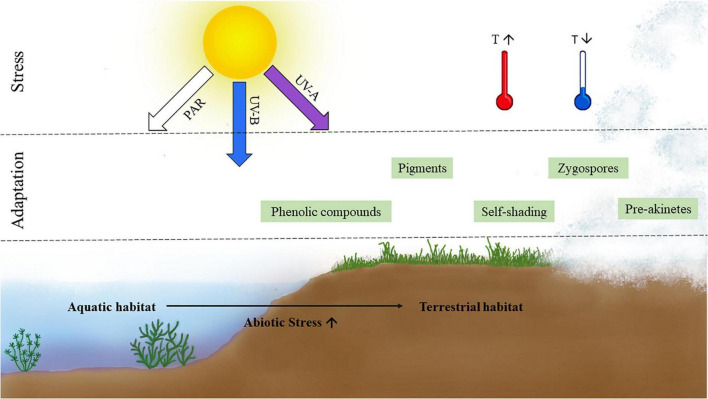
Schematic representation of two major abiotic stresses accompanying terrestrial habitats and associated adaptation strategies of zygnematophycean green algae.

**FIGURE 2:**
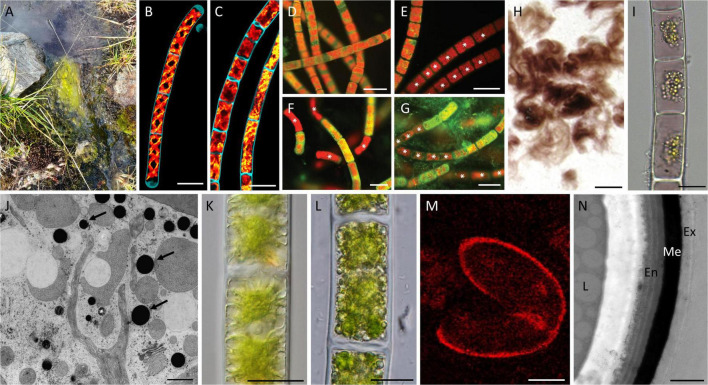
Effects and adaptation mechanisms of Zygnematophyceae to high radiation and temperature extremes. **(A)** Zygnematophyceae mat in semi-terrestrial habitat; **(B)** confocal micrographs of *Spirogyra* sp. maintained at 22°C; **(C)** confocal micrographs of *Spirogyra* sp. maintained at 37°C with altered plastids; **(D–G)** *Zygnema* sp. stained with 0.1% Auramine O, dead cells marked with an asterisk; **(D)** young culture (–2°C); **(E)** young culture (–10°C); **(F)** pre-akinetes (–20°C); **(G)** pre-akinetes (–70°C); **(H)** purple *Zygogonium ericetorum* filaments; **(I)** cells of *Z. ericetorum* with purple pigment in the vacuoles; **(J)** electron micrograph of *Zygnema* sp. exposed to PAR + UV-A showing electron-dense particles (arrows); **(K)** young *Zygnema* sp. cells after PAR + UV-A + UV-B (PAB) treatment; **(L)** pre-akinetes after PAB treatment; **(M)** RAMAN imaging of *Spirogyra* mirabilis zygospore showing aromatics in cell wall; **(N)** transmission electron micrograph of *Spirogyra* mirabilis zygospore cell wall showing three-layered structure and electron-dense middle layer; Abbreviations: En, endospore; Ex, exospore; Me, mesospore; and L, lipid. Scale bars **(B,C)** 100 μm; **(D–G)** 40 μm; **(H)** 1 cm; **(I,K,L)** 20 μm; **(J)** 1 μm; **(M)** 15 μm; **(N)** 1 μm. **(B,C)** Reprinted from de Vries et al. (2020); **(D–G)** Reprinted from Trumhová et al. (2019); **(H,I)** Reprinted from Aigner et al. (2013); **(J)** Reprinted from Pichrtová et al. (2013); **(K,L)** Reprinted from Holzinger et al. (2018); and **(M,N)** Reprinted from Permann et al. (2021a). All reprinted material was published under CC-BY license and is copyrighted by the authors.

The transition from aquatic to semi-terrestrial/terrestrial habitats entails many abiotic changes and challenges, which require special adaptation strategies (Buschmann and Holzinger, 2020; Figure 1). Organisms inhabiting terrestrial environments are often exposed to wider temperature extremes than in water (Hawes, 1989; Elster and Benson, 2004; Holzinger et al., 2009) and to higher levels of photosynthetically active radiation (PAR) and ultraviolet radiation (UV); however, living in water might also impose limitations, such as lower bicarbonate availability, which is overcome by the formation of efficient carbon concentration mechanisms such as the pyrenoids (Delwiche and Cooper, 2015; Harholt et al., 2016). Another major stressor in terrestrial habitats, water scarcity, potentially leading to desiccation and its negative consequences to green algae, has been reviewed elsewhere (Holzinger and Karsten, 2013; Becker et al., 2020). In contrast to terrestrial environments and perennial waterbodies, semi-terrestrial habitats are only periodically covered with water and/or have moist or merely humid conditions. Organisms in these habitats are regularly exposed to different levels of the aforementioned stresses (Figure 2A). Within Zygnematophyceae, the genera *Zygnema* and *Mougeotia* often inhabit such semi-terrestrial sites in the form of small rivulets or shallow pools (Pichrtová et al., 2018; Permann et al., 2021b). This class of green algae has a cosmopolitan distribution, including extreme environments such as the arctic and Antarctic regions (Holzinger et al., 2009; Kaplan et al., 2013; Pichrtová et al., 2013, 2014a,2014b). To survive under these harsh conditions, Zygnematophyceae have developed a plethora of avoidance mechanisms and adaptation strategies.

Importantly, today’s abiotic conditions are very different from the conditions about 500–600 million years ago (MYA), when the colonization of the terrestrial habitat might have taken place (Becker, 2013). The atmospheric carbon dioxide (CO_2_) concentration was much higher (∼4,000 ppm) than it is today (∼410 ppm), and the oxygen concentration was much lower (∼2%) compared to today’s ∼21%. The oxygen level drastically increased when plant biomass started to accumulate in the terrestrial habitat, and in turn this biomass functioned as a CO_2_ sink. Estimates put the global temperature at about 30°C and no polar ice caps were present ∼ 500 MYA (Climate.gov, 2020; Dahl and Arens, 2020). Finally, given the lower oxygen content in the atmosphere, any ozone layer was probably only thin, if present at all, allowing UV radiation to reach the surface at a much higher intensity. Thus, it seems safe to assume, that beside a lack of water, higher temperature and radiation were the major stressors affecting the ancestors of embryophytes during their transition to land.

A summary of abiotic stress tolerance in charophyte green algae was conducted by Holzinger and Pichrtová (2016) and reviews of desiccation stress in green algae were provided by Holzinger and Karsten (2013) and Becker et al. (2020). The present review focuses mainly on the effects of temperature and light stress and potential adaptation strategies in filamentous Zygnematophyceae. Important findings of the past three decades are gathered to comprise a comprehensive overview. This will improve our understanding of the prerequisites for terrestrialization and provide insights into the potential of this group for adaptation to future environmental changes occurring as a consequence of climate change.

## Temperature extremes are more pronounced in terrestrial environments

Algae occupying semi-terrestrial habits are exposed to more drastic temperature shifts, as water has a higher specific heat capacity (i.e., it gains and loses heat more slowly) than air. Heat stress can cause severe damage to cells at the level of protein activity and the fluidity of membrane lipids (Hu et al., 2020). Both acute heat stress and prolonged exposure to high temperatures can be harmful to algae. Studies of *Spirogyra* sp. originating from central Indiana showed that net photosynthesis was positive at temperatures up to 35°C (Graham et al., 1995). A lowland species (*Zygnema* sp. “Saalach,” SAG 2419, clade 2) and an alpine species (*Zygnema* sp. “Ellmaualm,” SAG 2418, clade 1) have also been shown to reach their maximum photosynthetic oxygen production at 35°C and 30°C, respectively, while oxygen production drastically decreased after the peak (Herburger et al., 2015). These data suggest that some members of the class Zygnematophyceae that are naturally exposed to higher temperatures are well adapted to these conditions. In contrast, *Zygnema* strains isolated from subpolar (*Zygnema* sp. I, SAG 2642, clade 2) and polar habitats (*Zygnema* sp. B, CCALA 976, clade 2) reached maximum photosynthetic oxygen production at comparatively lower temperatures of 20°C and 15°C, respectively, (Permann et al., 2022). Exposure of these strains to higher temperatures (25°C) for a period of 2 weeks also led to a decrease in photosynthesis. However, the subpolar and polar strains showed no change in their ability to dissipate absorbed energy, i.e., non-photochemical quenching (Permann et al., 2022). The different physiological responses to temperature stress within the same genus from different habitats suggest a possible relationship between high temperature tolerance and strain origin. Phylogenetic position had only a minor influence, as isolates from the same clade (clades according to Stancheva et al., 2012) showed large differences. Studies of *Cosmarium* sp. that found a relationship between physiological characteristics and their location of isolation support this hypothesis (Stamenković and Hanelt, 2012). It would be interesting to study more strains of tropical and subtropical origin, as these habitats reflect the conditions during terrestrialization.

A rather drastic and short-term increase in temperature (37°C for 24 h) applied to cultures of *Spirogyra pratensis* (MZCH 10213) and *Mougeotia* sp. (MZCH 240) led to phenotypic alterations of their plastids and changes in the expression level of genes that are known regulators of the stress response in land plants (e.g., upregulation of genes encoding for heat shock protein, aquaporin, Rossman-fold dehydrogenases; de Vries et al., 2020; Figures 2B,C), the latter strengthening their position as a sister lineage to embryophytes. Damage from such high temperatures can be avoided by mat formation; for example, mats of *Spirogyra* had a surface temperature of 32°C, while the water temperature below the mat was 15°C colder (Hillebrand, 1983). This would enable the lower and more-shielded cells to continue their photosynthesis activity even at high temperature levels. Genetic studies of *Mougeotia* sp. (MZCH 240) suggest a remodeling of the photosystem apparatuses (e.g., upregulation of genes associated with chlorophyll a/b-binding and light-harvesting) and accessory pigment composition after submergence of the filaments (Fürst-Jansen et al., 2021).

Cold stress is another enhanced challenge in today’s terrestrial habitats and a major inhibitor of growth and development in algae. However, cold stress might have been of only minor importance at the time of terrestrialization (see above). Nevertheless, as it is a common factor for many algae today, and the algal response in many aspects resembles that to desiccation, both leading to oxidative stress within the cell, we will briefly summarize the available data.

Oxidative stress caused by cold temperatures can be especially harmful, as enzymatic scavenging of free radicals may be less efficient (Han et al., 2009). Even a short exposure to subzero temperatures can be extremely damaging, especially to the plasma membrane, as it is the main site of freeze-thaw damage (Steponkus, 1984). *Zygnema* sp. obtained in a frozen state were able to recover immediately after thawing and transfer to fresh medium (Pichrtová et al., 2016b). Temperature treatments of 5 or 10°C both resulted in recovery of the photosynthesis activity, whereas culturing at 20°C increased the recovery rate. An Antarctic *Zygnema* sp. has also been reported to survive repeated freeze-thaw events (–4°C and 5°C) with no significant effects on its photosynthesis capacity (Hawes, 1990). In contrast, a prolonged exposure to –4°C resulted in decreasing photosynthesis rates.

However, not all members of Zygnematales seem to tolerate such freezing events, in a vegetative state (Steiner et al., 2021). Investigations of young vegetative cells of an arctic *Zygnema* sp. (MP2011Skan) strain showed an impairment of the physiological properties at –2°C, and no recovery when exposed to –8°C, due to frost damage (Trumhová et al., 2019; Figures 2D,E). Zygnematophyceae are still able to survive in the extreme arctic conditions through formation of so-called pre-akinetes, i.e., mature, storage compound-rich cells derived from vegetative filaments (Trumhová et al., 2019). Common resistant stages found in streptophyte green algae, such as parthenospores and aplanospores, however, have not been reported in polar regions (Stancheva et al., 2012; Trumhová et al., 2019). *Zygnema* inhabiting these inhospitable environments develop another asexually formed type of resting stages, termed pre-akinetes (Trumhová et al., 2019). Pre-akinete cells have smaller vacuoles, thicker cell walls, higher lipid contents, and smaller chloroplasts (Pichrtová et al., 2014a,b, 2016a,2016b). These cells also remain connected in filaments and can transform into vegetative cells, and *vice versa*, continuously (Herburger et al., 2015). Note that pre-akinetes are not necessarily converted into single celled “true akinetes,” covered with a dark, massive cell wall and no longer photosynthetically active. Pre-akinetes are beneficial in cold conditions, as they are able to survive extreme temperatures up to –20°C without ultrastructural changes (Trumhová et al., 2019; Figures 2F,G). The consequences of cold stress are, in part, similar to those of desiccation stress, as both lead to a decrease in intracellular water potential (Bisson and Kirst, 1995). Acclimation to water scarcity or corresponding adaptation strategies might also help to tolerate freezing events and *vice versa*.

In contrast to the aforementioned toleration strategies, some microalgae simply settle in habitats where they can avoid freezing events. *Micrasterias*, for example, has been shown to possess little frost tolerance, but is able to survive in waterbodies with snow cover (Steiner et al., 2021). This snow cover causes dark conditions and more-stable temperatures, preventing freezing events (Steiner et al., 2021). Further studies of *Micrasterias* revealed subcellular reorganization in the form of increased organelle contacts, after the cells were exposed to cold stress (Steiner et al., 2020). Especially, fusion of mitochondria in 3-dimensional networks was most prominently observed, which might enable the cells to maintain respiration even at low temperatures, by interconnecting mitochondrial respiratory chains.

Studies of the expression of genes responding to cold stress in *Spirogyra varians* revealed a gene, expressed at cold temperatures, that exhibited a shikimate-binding site (Han et al., 2009). The shikimate pathway plays a major role in biosynthesis of aromatic compounds, which are also heavily involved in protection from UV and reactive oxygen species, as described below (Han et al., 2009; Aigner et al., 2013; Pichrtová et al., 2013; Holzinger et al., 2018).

## Light stress as a new challenge in terrestrial environments

Another major abiotic stressor accompanying non-aquatic habitats is higher levels of light, both in the visible, PAR, and UV spectral ranges. The biologically important wavebands of UV-B (280–320 nm), UV-A (320–400 nm), and excessive PAR (400–700 nm) can potentially be damaging to the photosynthesis apparatus (Turcsányi and Vass, 2000; Vass et al., 2002).

Only relatively few studies have been performed exposing Zygnematophyceae to high PAR conditions or measuring electron transport rates and oxygen production in high-light conditions (Herburger et al., 2015; Thangaraj, 2015; Kakkou et al., 2016; Pierangelini et al., 2017). While some studies have suggested a higher resistance to photoinhibition and observed no damaging effects at 1,000 μmol photons m^–2^ s^–1^ (Thangaraj, 2015), other studies have suggested lower photoprotection capacity and less tolerance to high light intensity in *Zygnema* sp. (SAG 2419) (Pierangelini et al., 2017). In contrast, two *Klebsormidium flaccidum* isolates (one from acidic soil on a former mining site, one from an alpine soil crust at 2,400 m a.s.l.) from Austria *Klebsormidium flaccidum* (Klebsormidiophyceae) showed a broader photophysiological plasticity, due to a higher photoprotection capacity (Pierangelini et al., 2017). Recent transcriptomic data from *Klebsormidium nitens* (strain NIES-2285) exposed for 3 h to high light (1,500 μmol photons m^–2^ s^–1^) support this view and suggest that even LHC-like stress-related protein, known to be specific for Charophyta, contributes in combination with the regular streptophyte protection mechanisms (Serrano-Pérez et al., 2022).

In contrast to PAR, UV radiation is not vital for photosynthesis and is often the main source of radiation-induced damage in algae (Holzinger and Lütz, 2006). Especially solar UV radiation has been studied extensively over the last few decades, due to the increased level by human-caused changes in the stratospheric ozone layer (Björn et al., 1999). UV-B radiation can damage not only the photosynthesis apparatus, but respiration, growth, reproduction, and the molecular and ultrastructural integrity of photosynthetic organisms (e.g., Caldwell et al., 1998; Björn et al., 1999; Holzinger and Lütz, 2006). Zygnematophyceae are often exposed to high levels of UV radiation at high altitudes and latitudes.

Studies on the effects of high UV-B radiation on *Zygnema* isolates from a mountain lake (altitude 1,880 m a.s.l.) showed no impairment of the optimal quantum yield of PSII (Germ et al., 2009). In contrast, UV-B exposure of an Antarctic *Zygnema* strain led to a clear negative effect on PSII, paired with overall low levels of recovery (Prieto-Amador, 2016). *Zygnema* mats from arctic habitats exposed to high UVR:PAR ratios showed no damage or impairment of the photosynthesis capacity or changes to the ultrastructure (Holzinger et al., 2009). In accordance, exposure of different *Zygnema* strains (arctic, Antarctic, and temperate) to experimental UVR treatments in a sun simulator also did not result in severe changes to the photophysiological properties of PS II (Holzinger et al., 2018). Studies of polar *Zygnema* strains revealed varying levels of damage and responses to UV stress (Pichrtová et al., 2013).

## Protection strategies against light- and ultraviolet radiation stress

One simple but effective method for Zygnematophyceae to protect its cells from irradiation is the formation of mat-like sheets. The mats are a result of increased growth during wet periods, leading to substantial accumulation of biomass and subsequent water loss, packing the individual filaments tightly together. These layered structures, up to several millimeters thick, result in a self-shading effect of the bottom layer, as the upper cell layer often has thick, rigid cell walls (Holzinger et al., 2009). Self-shading has been observed in *Zygnema* sp. (Holzinger et al., 2009) as well as in *Zygogonium ericetorum* (Aigner et al., 2013) and might also result in low light adaptation of the photosynthesis apparatus in this situation (Herburger et al., 2015). It must be considered that more than one genotype may exist within a mat and therefore that the shading may be provided by different genera (e.g., Pichrtová et al., 2018; Trumhová et al., personal communication). In a recent metatranscriptomic study of arctic *Zygnema* sp., in the upper layer of a mat a drastic upregulation of transcripts was observed, targeting several biological processes and molecular functions related to protection of the photosynthesis apparatus and reduction of oxidative stress (Rippin et al., 2019). In polar regions, long periods of darkness might also affect plants, and a study with a polar *Cosmarium* strain showed a strong repression of transcripts associated with photosynthesis, photorespiration, and cell-wall development (Mundt et al., 2019).

Investigations of *Z. ericetorum* further showed that cells in the uppermost, sun-exposed layer exhibited a distinct dark-purple coloration of the vacuoles (Aigner et al., 2013; Figures 2H,I). This coloration is most likely caused by a complexation of polyphenolic moieties, such as gallic acid/ferric iron complex, and is lost upon culturing, presumably caused by the lack of UV exposure in the culture (Holzinger et al., 2010; Newsome and van Breemen, 2012; Aigner et al., 2013). The amount of UV-absorbing compounds was lower in the green morphotype of *Z. ericetorum* compared to the purple morphotype, which contained higher concentrations of hydrolyzable tannins and phenolic substances (Aigner et al., 2013). The purple morphotype was better protected against high irradiation than the green morphotype, indicating an additional photoprotective function of the pigment (Aigner et al., 2013; Herburger et al., 2016). Pigments with UV-screening ability can also be stored in extracellular mucilage, as shown in the newly described zygnematophycean genus *Serritaenia* (Busch and Hess, 2022). This specific strategy has not so far been found in other Zygnematophyceae and differs from the accumulation of reddish, water-soluble pigments in the vacuoles, as found, e.g., in *Z. ericetorum* (Aigner et al., 2013; Herburger et al., 2016). Formation of unpigmented mucilage sheets (pectin and hemicellulose) is widespread in Zygnematophyceae, and while they can enhance desiccation and freezing resistance (Knowles and Castenholz, 2008; Holzinger and Pichrtová, 2016; Herburger et al., 2019), they have long been suspected to additionally protect algal cells against short wavelengths, although no UV protection could be found (Lütz et al., 1997).

Arctic and Antarctic *Zygnema* strains, exposed to enhanced UV-A and UV-B (predominantly UV-A) to PAR ratio showed the accumulation of various phenolic-like compounds (Pichrtová et al., 2013; Figure 2J), a finding that was supported by a sun-simulation experiment (Holzinger et al., 2018; Figures 2K,L). Within Charophyta, accumulation of these UV-screening compounds has been reported only in Zygnematophyceae (Han et al., 2007; Remias et al., 2012a,b; Pichrtová et al., 2013; Holzinger and Pichrtová, 2016; Holzinger et al., 2018). Recent studies also discovered flavonoid-like pigments possessing UV-absorbing properties in *Penium margaritaceum*, which also occur in some species of Chlorophyta (Goiris et al., 2014; Jiao et al., 2020). In addition to the detection of phenolic compounds and flavonoids, recent data suggest the presence of parts of the phenylpropanoid pathway in streptophyte algae (Rieseberg et al., 2022; Serrano-Pérez et al., 2022). The phenylpropanoid pathway can be seen as central for numerous crucial land-plant structural components and stress responses, and has long been seen as a land-plant-specific adaptation (e.g., Dixon and Paiva, 1995; Thévenin et al., 2011; Lee et al., 2019; Rieseberg et al., 2022).

Phenolics also possess a high antioxidative potential (Goiris et al., 2012). Ionizing radiation (e.g., gamma radiation) significantly affects the antioxidant system, for example by increasing the concentration of phenolic compounds (Lee et al., 2012). However, treatment of an arctic *Zygnema* sp. with gamma irradiation caused physiological and proteomic changes only at the highest dose applied (Choi et al., 2015). Such ionizing radiation can lead to the formation of oxygen-derived free radicals, which can affect the morphology, anatomy, biochemistry, and physiology of algal cells (Lee et al., 2012). *Spirogyra varians* exposed to gamma irradiation, for example, showed an increase in antioxidant parameters and a higher content of total phenolics (Lee et al., 2012). Xanthophyll-cycle pigments have also been shown to reduce damage to the photosynthesis apparatus from light-induced oxidative stress (Latowski et al., 2011). Recent studies have shown that the xanthophyll cycle is operative in Zygnematophyceae (Feng et al., 2021) and that the xanthophyll-cycle pool size and the de-epoxidation state increase during exposure to UV radiation (Pichrtová et al., 2013; Holzinger et al., 2018).

## Sexual reproduction as a possible advantage in terrestrial environments

Formation of resistant resting stages is an effective strategy for adaptation to unfavorable environmental conditions. While Pichrtová et al. (2016b) showed that some arctic *Zygnema* sp. were able to survive an annual cycle entirely in a vegetative state, most young cells are limited in their capacity to tolerate abiotic stresses. Common resting stages are parthenospores, aplanospores, akinetes, and pre-akinetes, which are all formed asexually. During sexual reproduction, Zygnematophyceae perform a conjugation where two protoplasts fuse without the formation of flagellated male gametes. This process is potentially independent of liquid water, and formation of papillae can also occur under relatively dry conditions, suggesting that it provides an advantage under terrestrial conditions.

Successful induction of conjugation under laboratory conditions has been reported in only a few cases (e.g., Czurda, 1930; Ikegaya et al., 2012; Zwirn et al., 2013; El-Sheekh et al., 2018; Takano et al., 2019; Permann et al., 2021b; Pfeifer et al., 2022). While so far, no universal triggers and required environmental conditions for conjugation have been found in Zygnematophyceae, light intensity and nitrogen depletion are suspected to be important, especially in *Spirogyra* (Yamashita and Sasaki, 1979; Ikegaya et al., 2012; Zwirn et al., 2013; El-Sheekh et al., 2018; Takano et al., 2019; Permann et al., 2021b). Cultures of *Spiroygra* sp. have been shown to conjugate when placed in a nutrient-poor medium and exposed to a higher light intensity than used under standard culture conditions, i.e., 180 μmol photons m^–2^ s^–1^, which is about six times the light intensity of standard culture conditions (El-Sheekh et al., 2018; Takano et al., 2019; Permann et al., 2021a); however, UV radiation seems to inhibit sexual reproduction (Zwirn et al., 2013; El-Sheekh et al., 2018). Sexual reproduction leads to the formation of zygospores, which are suspected to endure abiotic stresses to a higher extent than their asexually formed counterparts (Permann et al., 2021a), mainly due to particularly complex zygospore walls. This may be another advantage when shifting to terrestrial habitats.

The thick zygospore cell wall is composed of three major layers, forming a protective structure against harsh environmental conditions (Poulícková et al., 2007; Permann et al., 2021a,b). Studies of *Mougeotia* and *Spirogyra* zygospores revealed that the inner and outer layers (endo- and exospore) contain mostly polysaccharides (Permann et al., 2021a,b). In contrast, the middle layer, the mesospore, shows a high electron density when viewed by TEM and contains lipids and a sporopollenin-like material (De Vries et al., 1983), possibly algaenan (Poulícková et al., 2007; Permann et al., 2021a,b; Figures 2M,N). Algaenan is a highly aliphatic non-hydrolyzable biomacromolecule, similar to cutans or suberans, which can prevent extensive water loss in higher plants (Blokker et al., 1998). As mentioned earlier, such mechanisms of adaptation to desiccation stress might also enhance tolerance to freezing events. Additionally, the phenolic compounds found in the mesospore might act as an efficient barrier against high radiation.

Interestingly, conjugation and/or the formation of zygospores rarely occur in the extreme environments of polar habitats (Holzinger et al., 2009; Pichrtová et al., 2018). Formation of the complex zygospore wall and the maturation process of the zygospores are cost-intensive processes that might not be affordable in such extreme environments.

The present review summarizes the current knowledge of tolerance and adaptation strategies of filamentous streptophyte green algae to temperature- and light stress, focusing on the past three decades. Only in the last few years have metabolomic (Holzinger et al., 2018; Rippin et al., 2019) and transcriptomic studies (e.g., Rippin et al., 2017, 2019; de Vries et al., 2018, 2020; Fürst-Jansen et al., 2021) become available in Zygnematophyceae, and these elucidated key targets of abiotic stresses. These mechanisms are of broad interest, with respect to terrestrialization, but also in view of human-induced global change. More studies on the potential of these algae and particularly the role of conjugation might shed light on the Embryophta and the persistence of streptophyte green algae over the next decades.

## Author contributions

AH conceived the review. CP wrote the draft manuscript and prepared the figures. BB and AH commented and edited the manuscript and provided financial resources. All authors agreed on the final version of the manuscript.

## Conflict of Interest

The authors declare that the research was conducted in the absence of any commercial or financial relationships that could be construed as a potential conflict of interest.

## Publisher’s Note

All claims expressed in this article are solely those of the authors and do not necessarily represent those of their affiliated organizations, or those of the publisher, the editors and the reviewers. Any product that may be evaluated in this article, or claim that may be made by its manufacturer, is not guaranteed or endorsed by the publisher.
